# Emotional intelligence as a predictor of job satisfaction: the mediating role of conflict management in nurses

**DOI:** 10.3389/fpubh.2023.1249020

**Published:** 2023-11-10

**Authors:** Iris Soriano-Vázquez, Mayela Cajachagua Castro, Wilter C. Morales-García

**Affiliations:** ^1^Unidad de Posgrado en Salud, Escuela de Posgrado, Universidad Peruana Unión, Lima, Peru; ^2^Escuela de Medicina Humana, Facultad de Ciencias de la Salud, Universidad Peruana Unión, Lima, Peru; ^3^Escuela de Posgrado, Universidad Peruana Unión, Lima, Peru; ^4^Facultad de Teología, Universidad Peruana Unión, Lima, Peru; ^5^Sociedad Científica de Investigadores Adventistas (SOCIA), Universidad Peruana Unión, Lima, Peru

**Keywords:** emotional intelligence, conflict management, job satisfaction, nurses, mediation

## Abstract

**Background:**

Emotional Intelligence (EI) has emerged as a pivotal factor in work effectiveness and well-being within the healthcare domain. Specifically, its significance is heightened in the nursing sector, where emotional and social demands are high. Additionally, job satisfaction and conflict management are recognized as vital predictors of patient care service quality. However, there is a dearth of research addressing the mediating role of conflict management in the relationship between EI and job satisfaction within a nursing context.

**Objective:**

This study aims to assess the mediating role of conflict management in the relationship between emotional intelligence and job satisfaction among nurses.

**Methods:**

The STROBE checklist for cross-sectional studies was followed. A cross-sectional and explanatory design was employed. Data were collected using self-reported questionnaires to measure emotional intelligence, conflict management, and job satisfaction. Structural Equation Modeling (SEM) was conducted to test the proposed hypotheses.

**Results:**

A total of 208 nurses aged between 18 and 65 years participated (*M* = 41.18, SD = 8.942). The findings confirmed a positive relationship between emotional intelligence and conflict management (*β* = 0.64, *p* < 0.001). Similarly, a positive relationship between conflict management and job satisfaction was observed (*β* = 0.37, *p* < 0.001). Moreover, conflict management was validated as a mediator in the relationship between emotional intelligence and job satisfaction (*β* = 0.77, *p* = 0.002).

**Conclusion:**

The study underscores the importance of emotional intelligence and conflict management as predictors of job satisfaction in nurses. The results suggest that interventions aimed at enhancing emotional intelligence might be an effective avenue for increasing job satisfaction, especially when conflict management strategies are integrated.

## 1. Introduction

In the realm of nursing, Emotional Intelligence (EI) has surfaced as a topic of mounting interest due to its impact on a range of job outcomes and its significance in professions marked by intense emotional demands and interactions with patients and colleagues (1, 2). EI has been linked with job performance, job satisfaction, organizational commitment, and the mental health of nurses (3, 4). In a field where job satisfaction is pivotal for efficiency and the quality of healthcare (5–7), conflict management emerges as a key component. Recognized as essential within nursing, conflict management has been closely tied with EI and job satisfaction (8–10). Conflicts in the workplace can adversely impact productivity, patient care, mental health of the healthcare staff, and the quality of services rendered (11, 12). Therefore, the ability to effectively manage conflicts among healthcare personnel becomes integral to enhancing job satisfaction and delivering timely, efficient, and patient-centered care (13).

Particularly in challenging contexts such as in Peru, job satisfaction among nurses is pertinent as they grapple with resource scarcity, excessive workloads, and high emotional burdens (14, 15). Additionally, cultural and organizational factors, including gender expectations and healthcare system hierarchies, influence the job satisfaction of Peruvian nurses (16, 17). In this respect, job satisfaction is shaped by both intrinsic and extrinsic factors (18), playing a crucial role in staff retention, work commitment, and patient care quality (19, 20). Addressing these challenges and promoting job satisfaction among nursing personnel necessitates viewing EI as a critical skill. EI not only equips nurses to handle stress better and make informed decisions, but also bolsters organizational commitment, reduces burnout and turnover rates, ultimately contributing to safer and higher-quality patient care (21). Given the heightened stress and emotional demands in healthcare, EI becomes pivotal in enhancing professional competence, mental well-being, and effective stress and conflict management, benefiting both healthcare professionals and patients (22).

The relationship between EI and job satisfaction in nursing is intricate and multifaceted. EI serves as a catalyst for other paramount factors such as empathy and communicative satisfaction, which in turn influence work well-being (23). In an environment where nurses encounter a broad spectrum of emotional experiences, EI becomes vital in balancing professional objectivity with empathy and care, thereby enhancing job satisfaction and organizational commitment (24). EI not only elevates overall clinical performance but can also be instrumental in retaining professionals within the nursing domain (25).

In this backdrop, conflict management stands out as a critical element in amplifying the relationship between EI and job satisfaction among nurses. Conflict management styles, like collaborative and integrative approaches, are indispensable in addressing emotional challenges and tensions within the medical care setting. Conflict resolution strategies, such as structured training and team-building, are pivotal tools in maintaining a healthy work environment and fostering job satisfaction (26). Moreover, EI in nurse managers not only enhances their conflict-handling capabilities but also aids in their professional development and commitment to healthcare management (27).

### 1.1. Literature review

#### 1.1.1. Emotional intelligence

Emotional intelligence (EI) is a multidimensional construct that refers to the ability to recognize, understand, use, and regulate emotions in oneself and others (28). EI has been linked to a number of advantageous outcomes in the workplace, including job performance, job satisfaction, mental health, and healthcare quality (3, 29). As a result, emotional intelligence may be crucial in the health services industry since nursing practitioners engage with customers more, must adhere to patient care, and bear a bigger emotional weight (3, 30).

EI and conflict management have been linked in studies conducted in a variety of settings, including the workplace and classroom (31–33). EI has been associated with a higher capacity in nursing to address and settle problems at work. As a result, nurses with higher EI are less likely to employ evasive or competitive strategies and more likely to handle conflict management techniques (9, 34). In this sense, nurses with high EI may be more sensitive to the emotions of others and, as a result, be able to foresee and resolve possible conflicts before they worsen. Additionally, being able to control one’s emotions can help nurses remain composed and objective in the face of conflict, which promotes problem-solving, the quest for win-win solutions, and improved job satisfaction (23, 35).

On the other hand, the relationship between emotional intelligence (EI) and job satisfaction has been studied in various contexts, highlighting its particular relevance in the field of nursing, a sector known for its high emotional and social demands. Studies show that elevated levels of EI are associated with greater job satisfaction and organizational commitment (36, 37). Furthermore, EI is especially critical in high-tension contexts, such as the COVID-19 pandemic, where it acts as a moderator in the effects of psychosocial risks, including burnout and psychosomatic issues (29, 38). Thus, emotional intelligence and job satisfaction emerge as critical predictors of occupational well-being, especially given the high rates of turnover and burnout in the nursing sector (23). This highlights the need for hospital policies that focus not only on technical efficiency but also on the development of emotional and communicative skills (23, 36).

#### 1.1.2. Job satisfaction

Job satisfaction is a complex, multifaceted construct that refers to an individual’s positive attitude and feelings about their job, and values the working conditions and associated rewards (39). Due to its effects on patient safety, staff retention, productivity, and performance, job satisfaction is significant in the lives of nurses (40, 41). Workplace circumstances, pay, social support from managers and coworkers, possibilities for professional growth, and decision-making autonomy are only a few of the variables that have an impact on job satisfaction (42–44). To deal with the problems nurses encounter at work and increase job satisfaction, emotional skills and tactics might be essential. EI may also serve as a stress-relieving buffer for nurses, resulting in higher work satisfaction (45–47). Because of the growing expectations and difficulties that nurses encounter in today’s healthcare system, work satisfaction is particularly important.

#### 1.1.3. Conflict management

Conflict, understood as a process involving two or more individuals with divergent interests, perceived threats to their needs, or concerns (48), is commonplace within the realm of nursing. These conflicts can occur between direct care nurses or with nursing managers. Underlying causes often relate to limited human resources, discrepancies in demands between nursing leaders, and interpersonal communication issues (49, 50). Conflict management emerges as a vital interpersonal skill, aiming at an individual’s ability to confront and resolve conflict situations (51). Its importance in the workplace cannot be underestimated since it directly impacts job satisfaction, performance, and employee well-being (52, 53). This management is particularly critical in nursing due to the interpersonal nature of the work, high emotional burden, and the need to collaborate with other healthcare professionals (54, 55). Nurses with heightened emotional intelligence tend to manage these conflicts more effectively, being able to anticipate and resolve disputes before they escalate (56, 57).

Furthermore, five primary styles to address conflicts have been proposed, according to Rahim (51). The “Integrating” style seeks collaborative solutions that satisfy all involved (49) and is especially favored in patient care situations, as observed among Peruvian nurses (49) and in intensive care units (58). On the other hand, the “Obliging or Accommodating” style seeks to maintain peace (59) and is less utilized by nurses (60), while the “Dominating” style is effective in critical situations requiring quick decisions (61). The “Avoiding” style involves evading the conflict, useful when time is needed or the conflict is trivial (62), although it’s not the predominant style among emergency nurses (63). Lastly, the “Compromising” style seeks middle-ground solutions and is commonly used in practice (64, 65). However, no style is superior in itself, as its selection depends on the context and relationship between parties. In this regard, it’s essential for nursing leaders to apply effective communication, positive leadership, and proper conflict management for a healthy work environment and to harness potential benefits of conflict, such as innovation and development (26, 66, 67).

**Figure 1:**
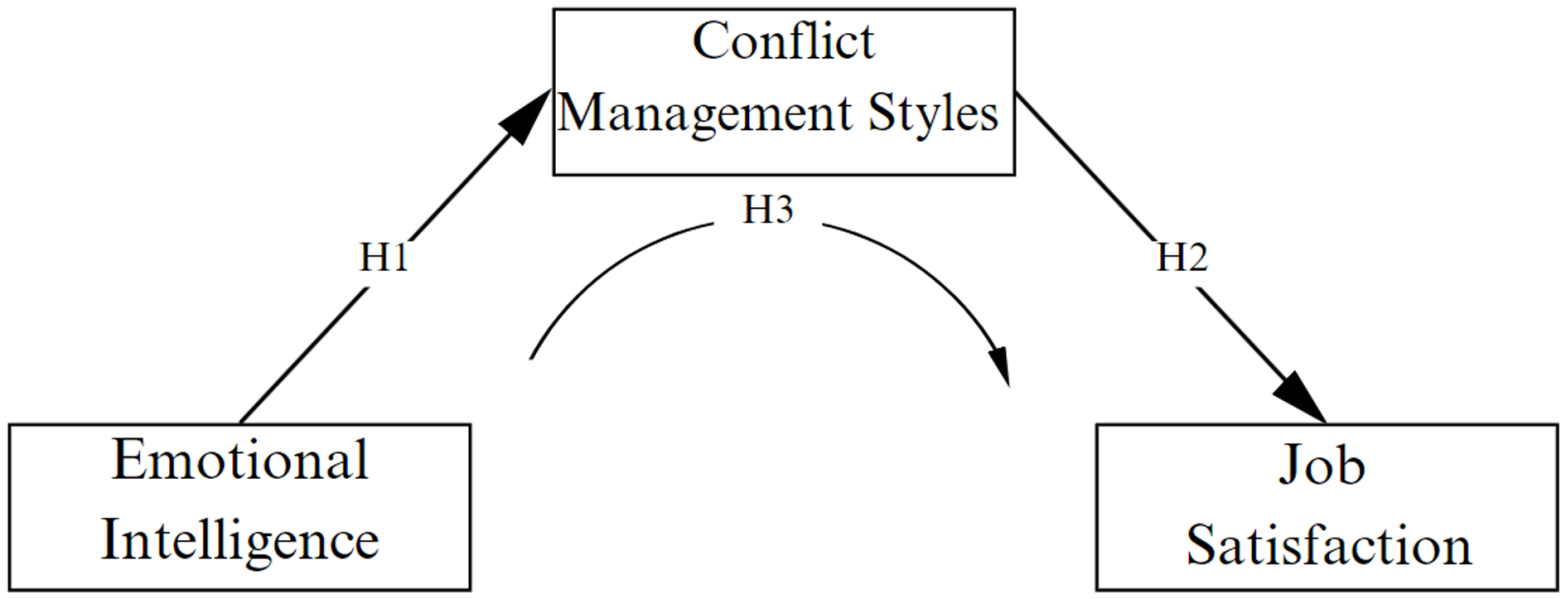
Theoretical model.

Few studies across different populations specifically address the mediating role of conflict management (68–70). Despite the growing evidence linking emotional intelligence, conflict management, and job satisfaction, research in the context of nursing has been reported in a theoretical manner up to this point (71). Given the pivotal role of conflict management in nursing practice and its potential connection with emotional intelligence and job satisfaction, it’s crucial to explore how conflict management might mediate this relationship. This could offer valuable insights for designing nursing interventions and training programs that address not only emotional skills development but also conflict resolution. Considering the arguments presented, the following hypotheses are proposed (Figure 1):

*H1:* There is a positive relationship between emotional intelligence and conflict management.

*H2:* There is a positive relationship between conflict management and job satisfaction.

*H3:* Conflict management mediates the relationship between emotional intelligence and job satisfaction.

## 2. Methods

### 2.1. Design and participants

The study was conducted under the guidelines proposed by STROBE. From the initial design, including the title (item 1), to details like funding (item 22), the stipulations set forth by this set of guidelines were followed (72). Within the parameters of this article, items 4 through 12 were specifically applied. These items are fundamental in guiding and structuring cross-sectional studies, ensuring their quality and transparency. For a more thorough review, please refer to Appendix A, where the complete STROBE checklist is broken down.

A cross-sectional and explanatory study was conducted, incorporating latent variables represented by a structural equation model (SEM) (73). A non-probabilistic sampling approach was used, in line with the consensus guidelines for measurement instruments in the healthcare sector (74). Inclusion criteria considered were: (1) employment in both critical and non-critical areas, with varying employment conditions that include outsourcing, contractual agreements, fixed-term, and appointment; (2) varying lengths of service, ranging from less than 1 year to more than 5 years. Exclusion criteria were: (1) retired or inactive nurses, (2) nurses on extended leave or absence during the study period. The sample size was determined using Soper’s software, which takes into account the number of observed and latent variables for SEM models. Through the anticipated effect size (*λ* = 0.3), statistical power levels (1 − *β* = 0.95), and desired probability (*α* = 0.05), the software recommended a sample size of 119 participants (75). However, the study ultimately included a total of 208 nurses, thereby exceeding the initial recommendations to enhance the robustness of the analysis.

### 2.2. Instruments

#### 2.2.1. Sociodemographic variables

Several sociodemographic variables were considered, including gender, marital status, employment status, length of time in the current service, and type of work area (critical or non-critical).

#### 2.2.2. Conflict management styles

The Spanish version (76) of the Rahim Organizational Conflict Inventory-II (ROCI-II), created by Rahim in 1983, was used. It consists of 28 evaluation items with five dimensions: integrating, dominating, avoiding, obliging, and compromising, and a 5-point Likert response scale (1 = Never to 5 = Always). Additionally, it showed adequate internal consistency through Cronbach’s Alpha for each of the dimensions, which were 0.70, 0.79, 0.72, and 0.88, respectively.

#### 2.2.3. Emotional intelligence

The Spanish version (77) of the Rotterdam Emotional Intelligence Scale (REIS) (78) was used. It consists of 28 items in four dimensions: (1) self-focused emotional appraisal, (2) other-focused emotional appraisal, (3) self-focused emotion regulation, and (4) other-focused emotion regulation, with a 5-point Likert response scale ranging from 1 (strongly disagree) to 5 (strongly agree). The internal consistency was adequate through Cronbach’s Alpha, being 0.86, 0.85, 0.80, and 0.86 for the dimensions, respectively.

#### 2.2.4. Job satisfaction

The Spanish version of the job satisfaction S20/23 (79) was used. It consists of 23 items and presents four dimensions: (1) relationship with supervision, (2) physical work space, (3) professional achievement, and (4) opportunity for training and decision-making. It presents 7 response alternatives: (1) Very Dissatisfied, (2) Quite Dissatisfied, (3) Somewhat Dissatisfied, (4) Indifferent, (5) Somewhat Satisfied, (6) Quite Satisfied, and (7) Very Satisfied. The scale showed adequate reliability through Cronbach’s Alpha of 0.92, 0.86, 0.78, and 0.73, respectively.

### 2.3. Procedure

Contact was established with the administrators of two selected hospitals, who not only approved the conduct of the study but also provided email addresses for the online administration of the survey. Data collection took place from February 14 to May 25, 2022, utilizing two methods: face-to-face and online. In the face-to-face method, measurement instruments were directly administered to nursing professionals in their respective work environments. Concurrently, in the online method, emails containing a link to the digital survey were sent out. It’s crucial to note that, prior to the administration of any instrument, participants were provided with a detailed explanation of the study’s objectives and purpose. This step was essential in obtaining informed consent from the participants.

### 2.4. Ethics statement

The study was reviewed and approved by the Institutional Ethics Committee of a Peruvian university (2023-CEUPeU-011) for hospital research. Ethical standards based on the Helsinki Declaration (80) were adhered to.

### 2.5. Statistical analysis

Descriptive statistics were calculated. Specifically, central tendency and dispersion measures were determined, such as the mean (M) and standard deviation (SD). Additionally, the measure of shape, skewness (A), was evaluated, where values within the range of ±2 for these measures suggest an approximately normal distribution (81, 82).

The analysis of the theoretical model of the study was carried out through structural equation modeling using the WLSMV estimator, appropriate for its robustness against deviations from inferential normality (83). The evaluation of the fit was carried out with the Comparative Fit Index (CFI), the Tucker Lewis Index (TLI), the Root Mean Square Error of Approximation (RMSEA), and the Standardized Root Mean Square Residual (SRMR). Values of CFI and TLI > 0.90 (84), RMSEA <0.080 (85), and SRMR <0.080 (86) were used. For the mediation analysis, the bootstrapping method was applied with 5,000 iterations and a 95% confidence interval (87). As for reliability analysis, the internal consistency method was used using the Cronbach’s alpha coefficient (α) expecting high magnitudes (> 0.70).

The structural equation modeling analysis was carried out with the “R” software in its version 4.0.5, using the “lavaan” library (88).

## 3. Results

### 3.1. Sociodemographic characteristics

A total of 208 nurses participated in the study, of which 88% were women and 12% were men, ranging in age from 18 to 65 years (*M* = 41.18, SD = 8.942). Additionally, 46.6% reported being married, 64.4% were tenured employees, 62% had been working in the service for more than 5 years, and 75.5% belonged to a non-critical area (Table 1).

**Table 1:** Sociodemographic information.

Characteristics		*n*	%
Sex	Female	183	88.0
	Male	25	12.0
Marital status	Single	82	39.4
	Married	97	46.6
	Free union	16	7.7
	Widowed	3	1.4
	Divorced	10	4.8
Employment condition	Outsourcing	4	1.9
	Contract	60	28.8
	Fixed term	10	4.8
	Permanent	134	64.4
Time working in the service	Less than 1 year	17	8.2
	1 year	20	9.6
	2 to 5 years	42	20.2
	More than 5 years	129	62.0
Area where you work	Critical	51	24.5
	Non-critical	157	75.5

### 3.2. Preliminary analysis

Table 2 presents the descriptive statistics for each variable including the mean (M), standard deviation (SD), and skewness (A). The correlations between the variables indicate that there is a significant positive correlation between conflict management styles, EI is (0.48, *p* < 0.001), and job satisfaction (0.31, *p* < 0.001). Also, the correlation between EI and job satisfaction (0.19, *p* < 0.001) was positive and significant.

**Table 2:** Descriptive statistics and correlations for the study variables.

Variable	M	DE	A	1	2	3
Conflict management styles	31.63	4.64	0.22	-		
Emotional intelligence	79.09	8.98	−0.39	0.48**	-	
Job satisfaction	95.26	23.38	−0.12	0.31**	0.19**	-

M, Mean; SD, Standard Deviation; A, Skewness; α, Cronbach’s Alpha. **p < 0.010.

### 3.3. Theoretical model analysis

**Figure 2:**
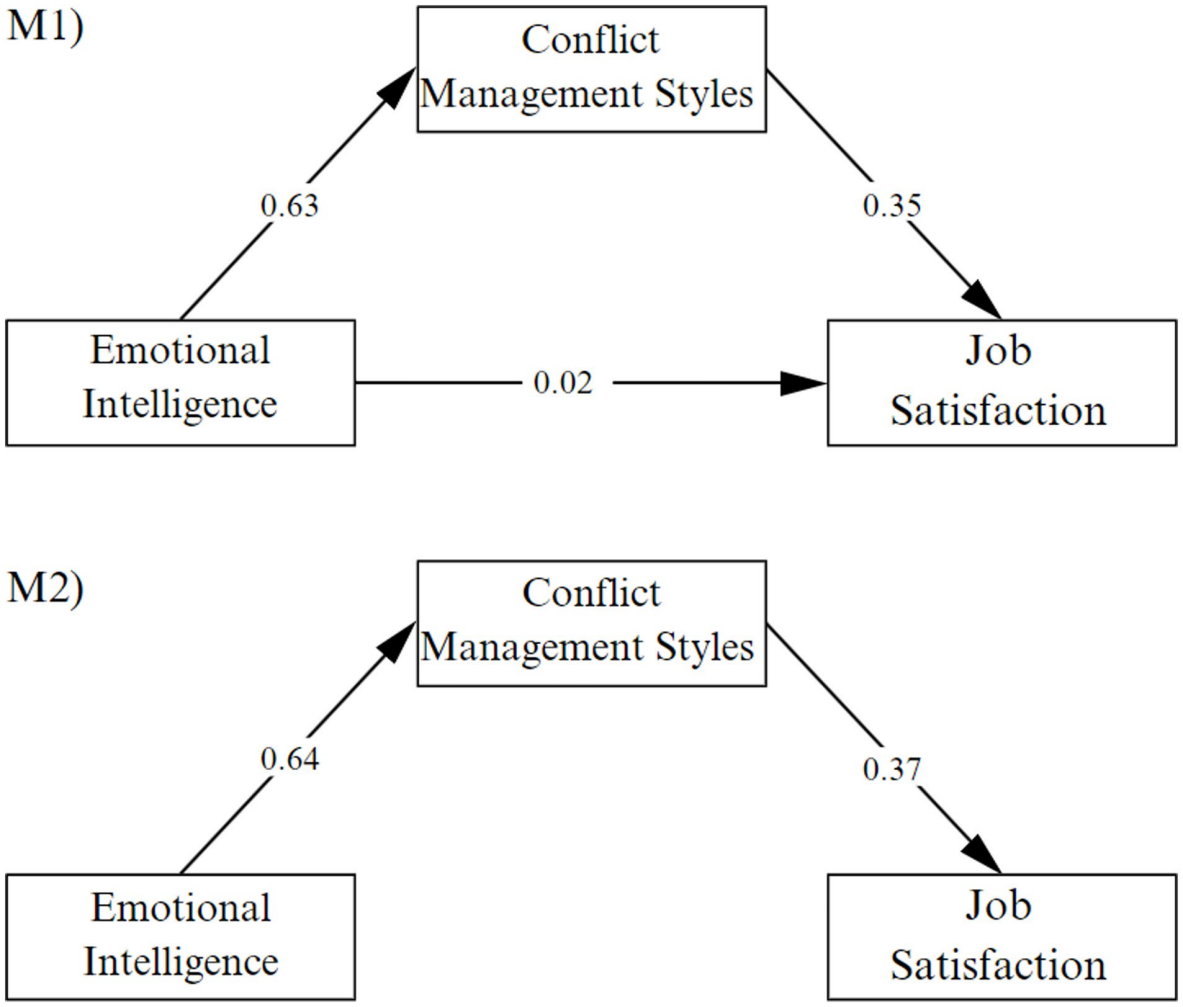
Structural model results: (M1) including direct effect and (M2) excluding direct effect.

A first model (M1) is made, in which the relationships between variables are incorporated, which obtained an adequate fit, *χ*^2^ = 1802.390, df = 1,213, *p* = 0.000, CFI = 0.95, TLI = 0.95, RMSEA = 0.05 (90% CI 0.04–0.05), SRMR = 0.08. However, due to the null effect value between emotional intelligence and satisfaction (*β* = 0.02 *p* > 0.5) and the considerations of parsimony criteria proposed in the model, a second model (M2) is chosen in which this relationship is restricted to zero, obtaining a good fit (*χ*^2^ = 1768.690, df = 1,214, *p* = <0.001, CFI = 0.95, TLI = 0.95, RMSEA = 0.05 [90% CI 0.04–0.05], SRMR = 0.08). In addition, H1 is confirmed, in which a positive relationship between emotional intelligence and conflict management is evidenced (*β* = 0.64, *p* < 0.001) and H2 in which conflict management is related to job satisfaction (*β* = 0.37, *p* < 0.001) (Figure 2).

### 3.4. Mediation model

For the mediation analysis, bootstrapping of 5,000 iterations was used and these results are shown in Table 3. The mediating role of conflict management in the relationship between emotional intelligence and job satisfaction is confirmed, *β* = 0.77, *p* = 0.002 (H3).

**Table 3:** Research hypotheses on indirect effects and their estimates.

				95%CI	
Hypothesis	Path in the model	*β*	*p*	LL	UL
Hypothesis 5a	Emotional Intelligence → Conflict Management → Job Satisfaction	0.77	0.002	0.40	0.23

## 4. Discussion

Emotional Intelligence (EI) is an increasingly relevant topic in both social and occupational settings, especially in professions characterized by high emotional demands and human interaction, such as nursing. The present research focuses on an emerging issue in healthcare, particularly in the nursing sector. It aims to analyze the mediating role of conflict management in the relationship between emotional intelligence and job satisfaction among Peruvian nurses. The results confirmed that conflict management effectively acts as a mediator between emotional intelligence and job satisfaction, thereby enhancing our understanding of the critical role emotional competencies play in workplace well-being. This research holds significant implications for the design of training programs and hospital policies, particularly in high-stress emotional and social settings like nursing. By highlighting the importance of emotional intelligence and conflict management, this study lays the groundwork for future interventions aimed at improving healthcare quality and the well-being of healthcare professionals.

The results of this study focused on the relationships proposed by the research model, providing evidence that confirms Hypothesis 1. This revealed a positive relationship between emotional intelligence (EI) and conflict management in nurses. This finding is consistent with previous research that has demonstrated a positive correlation between these two constructs (89, 90). A key aspect of this relationship is the intrapersonal and interpersonal skill that EI provides, allowing individuals to recognize and regulate their own emotions as well as those of others (21, 25). Adaptability and stress management, inherent traits of EI, facilitate conflict resolution (28). The ability to identify and understand emotions, for instance, can enable nurses to anticipate and avoid confrontational situations (35, 57). Moreover, emotional self-regulation, a subset of EI, can positively influence objective decision-making, promoting less polarized conflict resolutions (91). In workplace environments like healthcare services, the relevance of EI becomes even more pronounced. The nursing profession, with its high interaction with patients and emotional load, frequently faces challenges in conflict management. In this context, nurses with high levels of EI tend to employ more effective and less evasive or competitive strategies to address such conflicts (9, 34). These skills not only enhance conflict resolution but also positively influence job satisfaction, staff retention, and overall care quality (40, 41). However, it’s crucial to highlight the unique context of Peru, which, with its cultural and structural diversity, presents additional challenges for nurses (92). Skills derived from high EI are essential to tackle these specificities and the multiple sources of conflict that can arise due to divergences in objectives, demands, and interpersonal communication (49, 50). Organizations must recognize the importance of fostering EI, particularly in nursing. Since this profession continually interacts with human well-being, robust conflict management skills are essential to ensure optimal care and improve professionals’ well-being (51).

Hypothesis 2 was also confirmed, which evidenced a positive relationship between conflict management and job satisfaction in nurses. This is supported by previous studies that identified a similar connection across various professions (93, 94). Proper conflict management promotes a harmonious work environment and strengthens interpersonal relationships (95, 96), influencing nurses’ decisions to remain in their roles (97, 98). On the other hand, emotional intelligence (EI) plays a crucial role in effective conflict management in nursing. Nurses with high EI avoid evasive strategies and adopt more collaborative approaches (9, 34). These skills directly impact the creation of harmonious work environments, subsequently influencing job satisfaction (8). Moreover, EI has proven to be a valuable tool during crises, such as the COVID-19 pandemic, mitigating job stress and burnout (29, 38). Nonetheless, it’s essential to acknowledge that job satisfaction in nursing is influenced by several factors, including working conditions, compensation, social support, and autonomy (42). Therefore, healthcare institutions are concerned about nurse retention, as factors like inefficient management can influence their decision to stay or leave their roles (99). Also, conflicts in nursing can arise from various factors, including resource limitations or communication issues (50). Additionally, various conflict management styles exist, and Peruvian nurses tend to favor a collaborative approach (49). A combination of high EI and a collaborative approach in conflict management might, therefore, be the key to improving job satisfaction in nursing.

Moreover, Hypothesis 3 was confirmed, in which conflict management mediates the relationship between emotional intelligence (EI) and job satisfaction. This finding emerges as a significant contribution to the body of research in the field of nursing. This three-way relationship not only broadens the understanding of the role of emotional intelligence in high emotional labor contexts but also highlights the importance of conflict management as a crucial mediator (9, 100). Professionals with well-developed EI tend to be more effective in conflict management, leading to greater satisfaction in their work environment (91, 101). In challenging situations, such as the recent COVID-19 pandemic, it has been demonstrated that EI can act as a protective shield against psychosocial risks, underlining its role in job well-being (29, 38). Past literature has explored the relationship between EI and other constructs, such as social support (102) and work engagement (103). However, the focus on conflict management in this study provides a fresh and essential perspective (31). It’s pertinent to stress that a satisfactory work environment is crucial for nurse retention, as unfavorable conditions can lead to high turnover rates (99, 104). The importance of policies prioritizing the development of emotional and communicative skills in this context is undeniable (36). Conflict, often arising from limited resources, opposing objectives, or issues in interpersonal communication, should not be seen as detrimental (50, 105). Instead, it can be viewed as an opportunity for growth and strengthening of healthcare teams (106). It’s important to consider that in specific regions, like Peru, nursing professionals may face unique cultural and organizational challenges that require specialized conflict management skills (92). Lastly, professional training can benefit from these insights by integrating conflict management techniques. However, it’s essential to adjust these strategies considering contextual variables, such as organizational culture (9, 92).

### 4.1. Limitations

This study possesses several limitations that should be kept in mind when interpreting its findings. Firstly, the sample of nurses used may not adequately represent the broader nurse population, constraining the ability to generalize the results to wider contexts. To address this limitation, future research should consider more diversified samples, including nurses from various specialties, with different levels of experience, and from diverse geographical regions. Moreover, the cross-sectional design of the study prevents the establishment of robust causal relationships between emotional intelligence, conflict management, and job satisfaction. A longitudinal design, tracking nurses over time, would be more suitable to understand the temporal and causal dynamics between these variables. The non-probabilistic sampling is another constraint, as it focuses the study on a specific geographical and professional context, further limiting its generalizability. It’s crucial to account for uncontrolled variables in this study, such as work experience, educational level, and organizational support. These factors, which were not adequately addressed, could significantly impact job satisfaction and conflict management, potentially acting as moderator or mediator factors in the relationship between emotional intelligence and job satisfaction. A significant limitation is the lack of examination into the leadership style of key figures in the nursing realm, such as managers and head nurses. This oversight might exclude critical aspects influencing work dynamics and nurses’ satisfaction. Another point worth noting is the use of instruments which, although probing conflict management, do so indirectly and rely on nurses’ perceptions, potentially introducing biases. Looking forward, research should explore specific interventions aimed at enhancing the competencies identified as critical in this study. Replicating the study in different cultural and organizational contexts will validate and broaden the applicability of the findings. Additionally, it would be beneficial to explore the role of other psychosocial variables that might influence these relationships, thus deepening the understanding of the studied phenomenon.

### 4.2. Implications

As highlighted by this study, the nursing field underscores the significant interconnection between Emotional Intelligence (EI), conflict management, and job satisfaction. Within this context, it has been revealed that EI not only plays a pivotal role on its own but also directly influences conflict management, which in turn acts as a mediating factor toward job satisfaction. Effective application of emotional intelligence fosters a more harmonious work environment, especially in a critical area like nursing. By recognizing and managing one’s own emotions and those of others, misunderstandings are minimized, cultivating a conducive work atmosphere. The significance of these findings is not solely academic; it has far-reaching practical and strategic applications. Healthcare institutions, recognizing the importance of these competencies, should promote training programs focused on enhancing EI and conflict management skills. In doing so, they are not just investing in improving staff job satisfaction but are indirectly raising the quality of care provided to patients. Specifically, for head nurses and the director of nursing, there’s an imperative need to lead with ethics and authenticity. Their positions bestow upon them an added responsibility to model and encourage a leadership style that fosters a positive and collaborative work environment. Training in these areas not only benefits these leaders in their managerial roles but also has a cascading impact on all the staff under their charge. On a broader scale, health authorities should consider incorporating these findings into their policies, especially those related to staff retention and well-being. By doing this, they are not only advocating for the well-being of healthcare professionals but also ensuring high-quality medical care for society. In conclusion, this study’s contribution to existing literature is invaluable, shedding light on the mediating role of conflict management between EI and job satisfaction. In the future, it would be relevant to replicate and expand this research in various geographical and cultural contexts, solidifying the universality of these findings and potentially enriching understanding even further in this area.

## 5. Conclusion

In conclusion, this study has demonstrated the importance of emotional intelligence and conflict management as predictors of job satisfaction in nurses. The findings indicate that conflict management mediates the relationship between emotional intelligence and job satisfaction, suggesting that emotionally intelligent nurses may experience greater job satisfaction in part due to their ability to effectively manage conflicts.

## Data availability statement

The raw data supporting the conclusions of this article will be made available by the authors, without undue reservation.

## Ethics statement

The studies involving humans were approved by the study was reviewed and approved by the Ethics Committee of the Universidad Peruana Unión (2023-CEUPeU-011). The studies were conducted in accordance with the local legislation and institutional requirements. The participants provided their written informed consent to participate in this study.

## Author contributions

IS-V, MC, and WM-G participated in the conceptualization, validation, formal analysis and research, were in charge of the methodology and software, commissioned data and resource conservation, and handled first draft writing, review, editing, visualization, and supervision. All authors have read and approved the final version of the manuscript.
